# Contextual Factors of Face Mask Wearing During the COVID-19 Pandemic and Recommendations to Increase Compliance Rate

**DOI:** 10.21315/mjms2022.29.1.15

**Published:** 2022-02-23

**Authors:** Kim Hoe Looi

**Affiliations:** School of Economics and Management, Xiamen University Malaysia, Selangor, Malaysia

**Keywords:** face mask wearing compliance rate, demographic factors, personal hygiene factors, online behavioural factors, Malaysia

## Abstract

Community-wide face mask wearing is recognised as an effective non-pharmaceutical defence against infection by the severe acute respiratory syndrome coronavirus 2 (SARS-CoV-2), the causative virus of coronavirus disease 2019 (COVID-19) pandemic. However, few studies have identified contextual factors of face mask wearing during the COVID-19 pandemic. This study aims to identify relationships between demographic factors, personal hygiene factors, online behavioural factors and face mask wearing by Malaysian adults during the COVID-19 pandemic. Data were collected via an online survey questionnaire and analysed with Statistical Package for Social Sciences version 26. Non-availability of personal protective equipment (PPE) as well as fewer social media hours and fewer hours of browsing information related to the COVID-19 pandemic were identified as factors related to low compliance rate of face mask wearing by some Malaysian adults. This study advances contextual understanding of face mask wearing by specific groups during the COVID-19 pandemic and puts forth several recommendations to increase face mask wearing compliance rate.

## Introduction

After more than one year of ravages inflicted by the coronavirus disease 2019 (COVID-19) pandemic – caused by the severe acute respiratory syndrome coronavirus 2 (SARS-CoV-2) — pharmaceutical intervention involving vaccination has begun in many countries. Despite the availability of vaccines, access to vaccines in some countries remains an obstacle to eradicate the COVID-19 pandemic. Thus, non-pharmaceutical interventions at the individual level such as wearing face masks, personal hygiene (e.g. frequent washing of hands) and other physical barriers (e.g. physical distancing) are still paramount. Many public health agencies such as the US Centers for Disease Control and Prevention and the World Health Organization (WHO) recommend wide compliance with face mask wearing in public settings as part of a comprehensive infection prevention and control (IPC) strategy to reduce transmission of SARS-CoV-2 (1–3). Community-wide face mask wearing is not a new non-pharmaceutical intervention. Dr Lien Teh Wu, a Malayan epidemiologist, pioneered the development of surgical face mask and encouraged medical staff and the public to wear masks during the Manchurian plague of 1910–1911 (4).

Some scholars argue that there is little to lose and potentially much to gain from wearing a face mask (5). Wearing a face mask proffers various benefits as masks are cheap and easy to use, have strong sustainability, and good for health and economy (5, 6, 7, 8, 9, 10). Increasing scientific evidence supports the effectiveness of community-wide face mask wearing for the prevention and source control to reduce transmission of SARS-CoV-2 (3, 6, 7, 9, 10, 11, 12, 13, 14). However, the effectiveness of face masks is contingent upon community-wide adoption, high compliance rate most of the time and correct wearing of face masks (5, 8). Additionally, wearing of face masks in conjunction with other non-pharmaceutical interventions is critical for greater effectiveness in reducing transmission of SARS-CoV-2 (3, 6, 9, 10).

Despite their effectiveness, there is heterogeneity in face mask wearing (14, 15) due to various contextual factors, such as demographics, availability and cost of face mask, and culture. Several studies suggest that gender shapes face mask wearing adherence, with males less likely than females to wear a face mask (14, 15). Ethnicity is another factor that has affected face mask wearing adherence during the COVID-19 pandemic (5, 7, 10, 11, 12). Hearne and Niño (14) found that face mask wearing patterns in the U.S. during the COVID-19 pandemic were differently shaped by gender, ethnicity and interactions between these factors. Older individuals are slightly less likely to wear face masks compared to younger individuals (17). A recent study suggests that social media usage may be useful in predictive models of human behaviour during a pandemic (18).

Future research should examine person-context interactions in different geographic locations and cultural contexts to better predict face mask wearing during the COVID-19 pandemic (19). Understanding contextual factors of face mask wearing is important to increase face mask wearing compliance rate during the ongoing and future pandemics. As such, this study aims to identify relationships between demographic factors, personal hygiene factors, online behavioural factors and face mask wearing by Malaysian adults during the COVID-19 pandemic.

## Methods

This study has a cross-sectional design. The survey questionnaire was adopted from a previous study by Dai et al. (20) and was offered in the three major languages of Malaysia (Malay, English and Mandarin). Links to the questionnaire were distributed via popular social media platforms (e.g. WhatsApp and Facebook Messenger) and email. Online survey questionnaires are a safe and feasible way of collecting data during the COVID-19 pandemic (21).

To minimise response biases, standard survey approaches were followed (22) such that there was no social pressure to influence responses, no questions that would provoke defensiveness or threaten esteem and no payoff or cost for particular responses. Participation in this survey was voluntary and respondents could opt out at any time. Moreover, respondents were assured anonymity and confidentiality of their responses. All respondents consented to participate in this online survey by clicking the ‘Next’ button to complete the online questionnaire.

Data collected include respondents’ demographic characteristics (ethnicity, gender, age and number of children under 18 years old living in the same household), personal hygiene factors (frequency of washing hands and availability of PPE) and online behavioural factors (hours spent per day on internet, social media and browsing information related to the COVID-19 pandemic on social media). The dependent variable of frequency of face mask wearing was measured using three levels: low (wearing a face mask in public places less than 50% of the time); moderate (wearing a face mask in public places about 50% of the time) and high (wearing a face mask in public places more than 50% of the time).

Data were analysed with Statistical Package for Social Sciences (SPSS) version 26. Multinomial logistic regression was used to analyse factors associated with frequency of face mask wearing.

## Results

Table 1 depicts descriptive statistic of the respondents. Table 2 depicts hours spent per day on internet, social media and browsing information related to the COVID-19 pandemic on social media.

The multinomial logistic regression model is significant (*P* < 0.01). Likelihood ratio tests suggest that, overall, availability of PPE (*P* < 0.01), hours spent on social media (*P* < 0.01) and browsing information related to the COVID-19 pandemic (*P* < 0.05) were significantly related to frequency of face mask wearing (Table 3).

Compared with the high compliance rate group, the low compliance rate group had substantially lower access to PPE, followed by less exposure to social media, but members of this group were positively influenced by information related to the COVID-19 pandemic (Table 4). Meanwhile, compared with the high compliance rate group, the medium compliance rate group also lacked PPE, but the lack of access was less severe than that for the low compliance rate group (Table 4).

## Discussion

This study empirically identified that lack of PPE, fewer social media hours and fewer hours of browsing information related to the COVID-19 pandemic were associated with low compliance rate for face mask wearing by some adults in Malaysia. As opposed to investigating the general population (i.e. a shotgun approach), this study advanced contextual understanding of face mask wearing by specific groups during the COVID-19 pandemic. The divergent findings compared with prior studies on the influence of gender, ethnicity and age on face mask wearing underscores the importance of context in research concerning face mask wearing, as was also discussed in the Introduction section.

The benefits proffered by wearing face mask are subject to the availability and affordability of face masks over a prolonged period. Specifically, face mask expenses may further add to financial burdens experienced by low-income group during the COVID-19 pandemic. To increase community-wide face mask wearing compliance rate as part of a comprehensive strategy of infection prevention and control to reduce transmission of SARS-CoV-2, results of this study indicate that a two-pronged action plan involving free face mask use of social media platforms to provide reminders about face mask wearing and information related to the COVID-19 pandemic targeted at the low compliance rate group could be beneficial.

## Conclusion

This study has several limitations that should be addressed by future research. First, face mask wearing was measured at only a single time point during the pandemic. Second, given the contextual factors of face mask wearing, the findings here are not generalisable. As such, future research should investigate factors associated with face mask wearing in different countries and cultural settings for tailored non-pharmaceutical interventions.

Face masks were used during the Manchurian plague of 1910–1911. The WHO warns that more lethal viruses will emerge in the future (23). As such, face masks will continue to play an important role in future pandemics before new vaccines are widely available.

## Figures and Tables

**Table 1:** Descriptive statistic

Variable	Category	Frequency	Percent
Ethnicity	Malay	348	49.2
	Chinese	234	33.1
	Indian	41	5.8
	Natives of Sabah and Sarawak	85	12.0
	Total	708	100.0
Gender	Male	345	48.7
	Female	363	51.3
	Total	708	100.0
Age bracket (years old)	18–29	101	14.3
	30–39	213	30.1
	40–49	200	28.2
	50–59	159	22.5
	≥ 60	35	4.9
	Total	708	100.0
Number of children	None	335	47.3
	One	119	16.8
	Two	112	15.8
	Three	83	11.7
	Four	41	5.8
	Five	14	2.0
	Six or more	4	0.6
	Total	708	100.0

**Table 2:** Hours spent per day on internet, social media and browsing information related to the COVID-19 pandemic on social media

	Mean	Standard deviation
Internet	7.6	3.9
Social media	5.1	3.7
Browsing information related to the COVID-19 pandemic	2.5	2.5

**Table 3:** Likelihood ratio tests

Effect	Model fitting criteria	Likelihood ratio tests		
	
	−2 log likelihood of reduced model	Chi-squared	df	Sig.
Intercept	316.137	5.012	2	0.082
Ethnicity	312.370	1.245	2	0.537
Gender	311.498	0.373	2	0.830
Ethnicity X gender	312.200	1.075	2	0.584
Age	311.538	0.413	2	0.813
Number of children	312.909	1.784	2	0.410
PPE	340.195	29.070	2	0.000
Washing hands	316.782	5.657	2	0.059
Internet	311.232	0.108	2	0.948
Social media	324.126	13.001	2	0.002
Browsing information related to the COVID-19 pandemic	317.446	6.321	2	0.042

Note: The dependent variable is the frequency of face mask wearing; X denotes interaction between two variables

**Table 4:** Parameter estimates

Face mask wearing compliance rate^a^		Beta	Std. error	Wald	df	Sig.
Low compliance rate	Intercept	3.553	1.603	4.909	1	0.027
	Ethnicity	0.773	0.712	1.180	1	0.277
	Gender	0.622	1.031	0.364	1	0.546
	Ethnicity X gender	−0.333	0.452	0.542	1	0.462
	Age	−0.015	0.025	0.372	1	0.542
	Number of children	0.046	0.167	0.077	1	0.781
	PPE	−1.145	0.251	20.873	1	0.000
	Washing hands	−0.339	0.198	2.935	1	0.087
	Internet	0.021	0.073	0.079	1	0.778
	Social media	−0.406	0.144	7.964	1	0.005
	Browsing information related to the COVID-19 pandemic	0.332	0.141	5.567	1	0.018
Medium compliance rate	Intercept	0.840	1.592	0.278	1	0.598
	Ethnicity	0.314	0.843	0.139	1	0.709
	Gender	0.139	1.019	0.019	1	0.892
	Ethnicity X gender	−0.457	0.622	0.539	1	0.463
	Age	0.004	0.024	0.024	1	0.877
	Number of children	0.196	0.144	1.840	1	0.175
	PPE	−0.699	0.221	10.000	1	0.002
	Washing hands	−0.292	0.167	3.063	1	0.080
	Internet	0.014	0.070	0.038	1	0.846
	Social media	0.067	0.069	0.957	1	0.328
	Browsing information related to the COVID-19 pandemic	−0.064	0.107	0.364	1	0.546

Note:

a Reference group is high face mask wearing compliance rate;

X denotes interaction between two variables

## References

[1] Centers for Disease Control and Prevention Cover your mouth and nose with a mask when around others [Internet].

[2] World Health Organization Rational use of personal protective equipment for coronavirus disease (COVID-19) and considerations during severe shortages. Interim guidance, 6 April 2020 [Internet].

[3] World Health Organization Advice on the use of masks in the context of COVID-19. Interim guidance, 5 June 2020 [Internet].

[4] Wong S Face mask pioneer who helped defeat a plague epidemic [Internet]. New Scientist.

[5] Greenhalgh T, Schmid MB, Czypionka T, Bassler D, Gruer L (2020). Face masks for the public during the COVID-19 crisis. Brit Med J.

[6] Abboah-Offei M, Salifu Y, Adewale B, Bayuo J, Ofosu-Poku R, Opare-Lokko EBA (2021). A rapid review of the use of face mask in preventing the spread of COVID-19. Int J Nurs Stud Adv.

[7] Babak J, Weekes MP, Matheson NJ (2020). COVID-19: should the public wear face masks?. Brit Med J.

[8] Cowling BJ, Zhou Y, Ip DKM, Leung GM, Aiello AE (2010). Face masks to prevent transmission of influenza virus: a systematic review. Epidemiol Infect.

[9] Eikenberry SE, Mancuso M, Iboi E, Phan T, Eikenberry K, Kuang Y (2020). To mask or not to mask: modeling the potential for face mask use by the general public to curtail the COVID-19 pandemic. Infect Dis Model.

[10] Ma Q, Shan H, Zhang H, Li G, Yang R, Chen J (2020). Potential utilities of mask-wearing and instant hand hygiene for fighting SARS-CoV-2. J Med Virol.

[11] Van Bavel JJ, Baicker K, Boggio PS, Capraro V, Cichocka A, Cikara M (2020). Using social and behavioural science to support COVID-19 pandemic response. Nat Hum Behav.

[12] Chen X, Ran L, Liu Q, Hu Q, Du X, Tan X (2020). Hand hygiene, mask-wearing behaviors and its associated factors during the COVID-19 epidemic: a cross-sectional study among primary school students in Wuhan, China. Int J Environ Res Public Health.

[13] Cheng VC, Wong SC, Chuang VW, So SY, Chen JH, Sridhar S (2020). The role of community-wide wearing of face mask for control of coronavirus disease 2019 (COVID-19) epidemic due to SARS-CoV-2. J Infect.

[14] Hearne BN, Niño MD (2021). Understanding how race, ethnicity, and gender shape mask-wearing adherence during the COVID-19 pandemic: evidence from the COVID impact survey. J Racial Ethn Health.

[15] Zhang SX, Looi KH, Li N, Wan X, Li L (2021). Individual-level heterogeneity in mask wearing during the COVID-19 pandemic in Malaysia. Am J Trop Med Hyg.

[16] Haischer MH, Beilfuss R, Hart MR, Opielinski L, Wrucke D, Zirgaitis G (2020). Who is wearing a mask? Gender-, age-, and location-related differences during the COVID-19 pandemic. PLoS ONE.

[17] Howard MC (2021). The relations between age, face mask perceptions and face mask wearing. J Public Health.

[18] Zhang SX, Graf-Vlachy L, Looi KH, Su R, Li J (2020). Social media use as a predictor of handwashing during a pandemic: evidence from COVID-19 in Malaysia. Epidemiol Infect.

[19] Howard MC (2021). Gender, face mask perceptions, and face mask wearing: are men being dangerous during the COVID-19 pandemic?. Pers Individ Differ.

[20] Dai H, Zhang SX, Looi KH, Su R, Li J (2020). Perception of health conditions and test availability as predictors of adults’ mental health during the COVID-19 pandemic: a survey study of adults in Malaysia. Int J Environ Res Public Health.

[21] Srivastav AK, Sharma N, Samuel AJ (2020). Impact of coronavirus disease-19 (COVID-19) lockdown on physical activity and energy expenditure among physiotherapy professionals and students using web-based open E-survey sent through WhatsApp, Facebook and Instagram messengers. Clin Epidemiol Glob Health.

[22] Hughes K (2019). How to prevent response bias when conducting surveys [Internet].

[23] Parkhill M (2020). When it comes to pandemics, COVID-19 might not be ‘the big one’: WHO [Internet]. CTV News.

